# Applications of the Cholesterol Metabolite, 4β-Hydroxycholesterol, as a Sensitive Endogenous Biomarker for Hepatic CYP3A Activity Evaluated within a PBPK Framework

**DOI:** 10.3390/pharmaceutics16101284

**Published:** 2024-09-30

**Authors:** Aneesh V. Karkhanis, Matthew D. Harwood, Felix Stader, Frederic Y. Bois, Sibylle Neuhoff

**Affiliations:** Certara UK Limited, Certara Predictive Technologies, Level 2-Acero, 1 Concourse Way, Sheffield S1 2BJ, UK; matthew.harwood@certara.com (M.D.H.); felix.stader@certara.com (F.S.); frederic.bois@certara.com (F.Y.B.); sibylle.neuhoff@certara.com (S.N.)

**Keywords:** drug–drug interactions, 4β-hydroxycholesterol, cholesterol, endogenous biomarker, PBPK, CYP3A4, Simcyp Simulator

## Abstract

**Background/Objectives:** Plasma levels of 4β-hydroxycholesterol (4β-OHC), a CYP3A-specific metabolite of cholesterol, are elevated after administration of CYP3A inducers like rifampicin and carbamazepine. To simulate such plasma 4β-OHC increase, we developed a physiologically based pharmacokinetic (PBPK) model of cholesterol and 4β-OHC in the Simcyp PBPK Simulator (Version 23, Certara UK Ltd.) using a middle-out approach. **Methods:** Relevant physicochemical properties and metabolic pathway data for CYP3A and CYP27A1 was incorporated in the model. **Results:** The PBPK model recovered the observed baseline plasma 4β-OHC levels in Caucasian, Japanese, and Korean populations. The model also captured the higher baseline 4β-OHC levels in females compared to males, indicative of sex-specific differences in CYP3A abundance. More importantly, the model recapitulated the increased 4β-OHC plasma levels after multiple-dose rifampicin treatment in six independent studies, indicative of hepatic CYP3A induction. The verified model also captured the altered 4β-OHC levels in CYP3A4/5 polymorphic populations and with other CYP3A inducers. The model is limited by scant data on relative contributions of CYP3A and CYP27A1 pathways and does not account for regulatory mechanisms that control plasma cholesterol and 4β-OHC levels. **Conclusion:** This study provides a quantitative fit-for-purpose and framed-for-future modelling framework for an endogenous biomarker to evaluate the DDI risk with hepatic CYP3A induction.

## 1. Introduction

**Figure 1:**
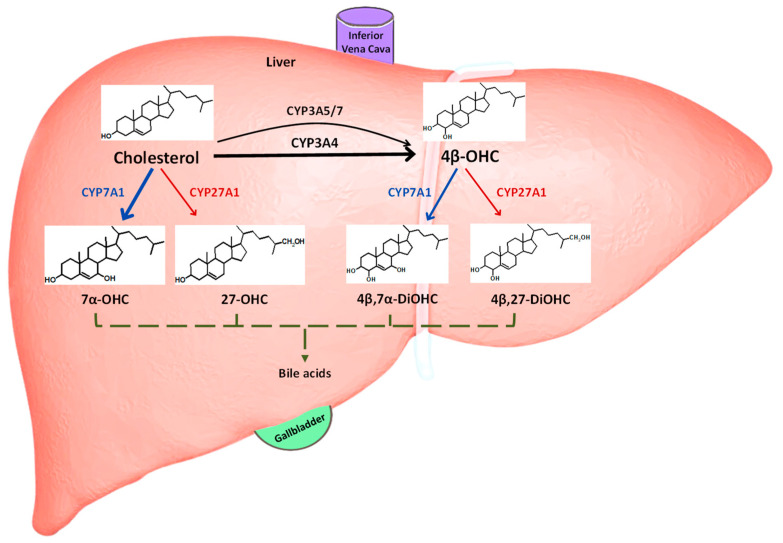
Hepatic elimination routes of cholesterol and 4β-OHC. The schematic shows 4β-OHC is generated predominantly via CYP3A4 from hepatic cholesterol, while CYP3A5 and CYP3A7 play a minor role. Cholesterol is either synthesized de novo in the liver or derived from diet. Both cholesterol and 4β-OHC are also independently metabolized by CYP7A1 (classic) and CYP27A1 (alternative) pathways to their respective dihydroxy metabolites and eventually bile acids, which are then released via bile into the gallbladder. 7α-OHC: 7α-hydroxycholesterol; 27-OHC: 27-hydroxycholesterol; 4β, 7α-DiOHC: 4β, 7α-dihydroxycholesterol; 4β, 27-DiOHC: 4β, 27-dihydroxycholesterol.

Identifying drug–drug interactions (DDIs) mediated via cytochrome P450 (CYP) pathways is important as approximately 50% of marketed drugs are metabolized by CYPs. Of these, CYP3A4 is the most prominent, metabolizing around 40% of prescribed drugs; thus, for nearly 20% of drugs on the market, CYP3A4 is their major elimination pathway [1]. Traditionally, CYP3A4-mediated DDIs are investigated clinically using index substrates such as midazolam or triazolam and perpetrators such as itraconazole for inhibition and rifampicin for induction of CYP3A. However, having a better estimation of individual responses to such perpetrators would allow us to establish personalized medicine by optimizing drug dosages. Hence, there is a desire to identify biomarkers that are reflective of CYP3A DDIs in vivo. 4β-Hydroxycholesterol (4β-OHC) is an endogenous oxidized metabolite of cholesterol generated by CYP3A4 and, to a minor extent, CYP3A5 and CYP3A7 in the liver [2] (Figure 1). Although CYP3A is highly expressed in the intestine, prior studies have demonstrated that its contribution to plasma levels of 4β-OHC is negligible [3]. In fact, plasma 4β-OHC levels are primarily reflective of the hepatic CYP3A activity [3]. Cholesterol and 4β-OHC are also metabolized by CYP7A1 and CYP27A1 to their respective oxidized metabolites (Figure 1), which form the backbone of bile acid synthesis pathways [4]. In vitro studies indicate that CYP7A1 preferentially metabolizes cholesterol with an about two-fold higher turnover rate than 4β-OHC [4]. Hence, metabolism by CYP7A1 is a rate-determining step in the clearance of 4β-OHC, resulting in a relatively long elimination half-life and a long time for stabilization of plasma concentrations [5]. As a result, 4β-OHC may be a more valuable measure of CYP3A induction than of reversible CYP3A inhibition.

Several clinical studies in healthy subjects have shown perturbation of 4β-OHC levels in response to strong CYP3A inhibitors (atazanavir/ritonavir, ketoconazole, and itraconazole) [6,7,8] or inducers (rifampicin, efavirenz, and carbamazepine) [2,7,8], which highlight its role as a potential clinical biomarker for CYP3A activity. There is considerable interest in estimating the 4β-OHC levels in patients using predictive mechanistic models to approximate the impact of novel drugs on CYP3A activity. For example, Leil et al., developed a model to evaluate DDIs of 4β-OHC with ketoconazole or rifampicin [9]. Similarly, Ngaimisi et al., developed a single compartment enzyme turnover pharmacokinetic model of plasma cholesterol and 4β-OHC/cholesterol ratio to evaluate the CYP3A4 inductive effects of efavirenz in HIV patients [10]. Li et al. [11] further modified the Ngaimisi model to assess enasidenib-induced CYP3A activity. However, these models derive heavily from published clinical trials and cannot account for the impact of covariates such as sex, CYP450 polymorphisms, and disease status, thus limiting their predictive ability. In the last 10 years, in vitro-to-in vivo extrapolation (IVIVE) methods that are linked to physiologically based pharmacokinetic (PBPK) models have become common to predict CYP450-mediated DDIs [12]. PBPK models consider the complex interplay between physiological (systems) data and drug-related parameters, routinely generated in vitro, to predict the pharmacokinetics of drugs [13]. PBPK models are ideally suited to investigate PK in complex clinical scenarios involving specific populations, differing ethnicities, pharmacogenetic variations, and food effects. In this study, we developed a PBPK model to reproduce the clinically observed baseline 4β-OHC levels in healthy adult subjects. We then assessed our model performance in predicting DDIs with CYP3A inducers. We investigated the effects of ethnicity, sex, and diseases such as rheumatoid arthritis (RA) on baseline 4β-OHC levels. Finally, we simulated DDIs with CYP3A inducers in healthy and RA populations.

## 2. Materials and Methods

A parent/metabolite PBPK model was developed for cholesterol and 4β-OHC based on available physicochemical, in vitro experimental, and clinical data. Model performance was first verified by comparing the predicted baseline steady-state plasma concentrations of 4β-OHC in several ethnic populations to observed data. Next, the predicted extent of plasma 4β-OHC increase in response to strong CYP3A inducers such as rifampicin was compared to observed data from clinical DDI studies. The verified model was then applied to predict the 4β-OHC plasma levels in specific populations, such as those with CYP3A4/5 genotypes, inflammatory conditions like RA, and induction by other CYP3A inducers like carbamazepine.

### 2.1. Establishing 4β-OHC Baseline Plasma Concentrations across Populations by Meta-Analysis

We first aimed to establish the baseline 4β-OHC levels in a healthy population since recovering baseline levels is a key aspect of model development and verification and underpins the extent of CYP3A induction. We searched the PubMed and Certara Drug Interaction Solutions (CDIS, Certara Inc., USA, formerly Drug Interactions Database (DIDB, University of Washington)) databases for studies where 4β-OHC baseline levels were reported. Studies reporting values for healthy adult individuals (>18 years old) from different ethnicities were included, while studies in diseased populations or where only cholesterol/4β-OHC ratio was reported were excluded with some exceptions. For example, data from epilepsy patients was included in the meta-analysis, as epilepsy *per se* did not significantly impact hepatic CYP3A levels [2,14]. This was deduced from clinical studies where treatment of epilepsy patients with valproate or levetiracetam (both CYP3A non-inducers) had 4β-OHC levels comparable to healthy individuals [2,14], as opposed to carbamazepine (a CYP3A inducer), which showed marked elevation of 4β-OHC plasma levels [2]. Similarly, data from patients with resected colorectal adenomas and gallstones was included in the analysis. However, individuals with a prior history of cancer, liver disease, inflammatory bowel syndrome, etc. were excluded, as these diseases are expected to affect the baseline cholesterol and 4β-OHC levels [15,16]. Finally, a study involving patients who had undergone kidney transplantation was also included, as the samples were collected 3 months after the transplant surgery [17]. Clinical studies indicate that after about 2.5 months of transplant surgery, hepatic CYP3A activity is restored to baseline levels [14]; therefore, it was assumed that 4β-OHC plasma levels reflected baseline upon sample collection. We also collated data for cholesterol baseline levels from the published literature with the same inclusion/exclusion criteria.

From each study, the sample size, mean, and standard deviation (SD) of 4β-OHC plasma concentrations were recorded, and the weighted mean and coefficient of variation (CV, %) were calculated. Analysis was performed using data from different ethnic groups such as Caucasian, Asians (comprising of Korean, Japanese subjects), Koreans, and African/African Americans. Additionally, data was collected for males and females in different ethnic populations.

### 2.2. PBPK Model Development

**Figure 2:**
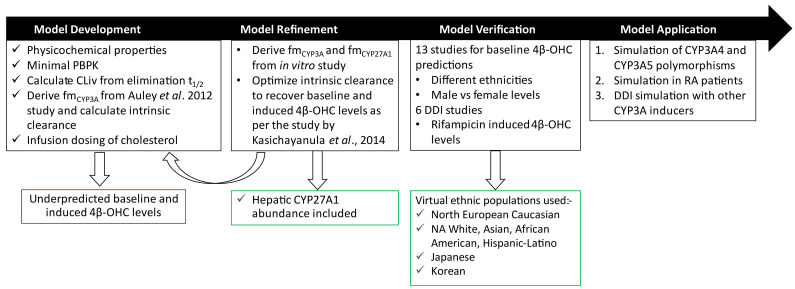
Workflow for cholesterol and 4β-OHC PBPK model development and verification. The model was initially developed using a bottom-up approach by incorporating physicochemical data and calculating CL_iv_ and ${fm}_{CYP3A}$ from Auley et al. [22]. However, this base model underpredicted the reported baseline 4β-OHC levels. The model was refined by optimizing CYP3A-specific intrinsic clearance of cholesterol as per the Kasichayanula et al. study [8]. The optimized model was verified with clinical studies where baseline 4β-OHC levels were reported in different ethnic populations and DDI studies with rifampicin as described in Tables S3 and S4, respectively. The verified model was further applied to prospectively assess its behaviour with respect to CYP3A4/3A5 polymorphisms, RA, and other CYP3A inducers. CLiv, intravenous clearance; DDI, drug–drug interaction; fm, fraction metabolized by specific enzyme; NA, North American; RA, rheumatoid arthritis.

The PBPK model was developed for cholesterol as the ‘parent’ (substrate moiety) and 4β-OHC as ‘primary metabolite’ (PM1) in Simcyp V23 (Certara UK Limited, Sheffield, UK) (Figure 2). The final input parameters for the cholesterol and 4β-OHC parts of the model are summarized in Table 1. Physicochemical parameters such as molecular weight (g/mol) and logP were obtained from PubChem or the Human Metabolome Database (HMDB). The blood-to-plasma ratio (B/P) for cholesterol was obtained from the literature, and it was assumed to be the same for 4β-OHC [18,19]. Fraction unbound in plasma for both compounds was predicted using an in silico tool developed previously [20]. Since apolipoprotein B forms a dominant component of cholesterol-rich lipoproteins in plasma, it was used as the representative plasma protein binding component [21].

#### 2.2.1. Distribution

In humans, the liver and intestine synthesize de novo about 70–80% and 10% cholesterol, respectively, while the remainder is derived from dietary sources [24]. The majority of the de novo synthesized cholesterol is further metabolized to bile acids or oxysterols, stored as cholesterol esters, and the remainder is bound to lipoproteins that are released into the systemic circulation [25]. In our model, we assumed that distribution of cholesterol to other tissues at steady state would have marginal impact on liver concentrations, and hence, we chose a minimal PBPK model to explain its distribution kinetics. Similarly, 4β-OHC is also primarily synthesized and metabolized in the liver, while a fraction is released in the systemic circulation. It was assumed that the distribution of 4β-OHC to other organs is minimal and hence we used a volume of distribution at steady-state (*V_ss_*) value of 0.05 L/kg (CV = 15%). The *V_ss_* value for cholesterol was also assumed to be low (0.1 L/kg, CV = 15%)). The liver over plasma partition coefficient (K_p_) was calculated from a preclinical study [23] (Table S1).

#### 2.2.2. Elimination

Cholesterol is metabolized to 4β-OHC by CYP3A4 and to a minor extent by CYP3A5 and CYP3A7 (Figure 1). Both cholesterol and 4β-OHC are also metabolized by CYP7A1 and CYP27A1. Since CYP27A1 is not currently available in the Simcyp Simulator, the functionality of CYP2J2 was used as a proxy for the CYP27A1, and the hepatic abundance of CYP27A1 was assigned to it in the model; a value of 41.93 pmol/mg microsomal protein was derived from a proteomics study, as described in Section S1 in the Supplementary Materials [26]. Due to a lack of hepatic abundance data for CYP7A1, elimination via this pathway was attributed to additional hepatic clearance by the human liver microsome (HLM).

Step 1: Calculation of CYP450-Specific Intrinsic Clearance of Cholesterol

The following workflow was used to determine the CYP450-specific intrinsic clearance of cholesterol (Figure 2):

The intravenous clearance (${CL}_{iv}$ in L/h) of cholesterol was calculated from its in vivo elimination half-life (*t_1/2_*) (mean 46.05 ± a SD of 16.58 days) [27,28] and *V_ss_* (described above) using Equation (1).

(1) $${CL}_{iv}=\frac{0.693\times V_{ss}\times W}{t_{\frac{1}{2}}}$$

where *V_ss_* is the volume of distribution at steady state (L/kg), W is the average human body weight of subjects in the studies (kg), and *t_1/2_* is the elimination half-life of cholesterol (h).

A reverse translation approach using the well-stirred liver model was applied to calculate the total hepatic metabolic intrinsic clearance (${CL}_{met,H}$) of cholesterol from ${CL}_{iv}$ using Equation (2).

(2) $${CL}_{met,H}=\frac{Q_{H}\times {CL}_{iv}}{{fu}_{p}\times (Q_{H}-\frac{{CL}_{iv}}{B/P})}$$

where ${CL}_{met,H}$ is the total hepatic metabolic intrinsic clearance (L/h), ${CL}_{iv}$ is the in vivo intravenous clearance (L/h), Q_H_ is the hepatic blood flow (L/h), B/P is the blood-to-plasma ratio, and ${fu}_{p}$ is the fraction unbound in plasma.

For CYP27A1, we determined the fraction of cholesterol it metabolizes ${fm}_{CYP27A1}$ and the intrinsic clearance of cholesterol by this pathway. In a previous in vitro study using recombinant CYP27A1, it was shown that 27% of cholesterol is metabolized by CYP27A1 [4]. We assumed this contribution to reflect the in vivo ${fm}_{CYP27A1}$ value. We determined the CYP27A1-specific whole organ hepatic metabolic intrinsic clearance (${CL}_{met,H,CYP27A1}$) and intrinsic clearance (CL_int_) per pmol of CYP27A1 (${{CL}_{int,}}_{u,H,CYP27A1}$), using Equations (3) and (4), respectively.

(3) $${CL}_{met,H,CYP27A1}={fm}_{CYP27A1}\times {CL}_{met,H}$$

(4) $${{CL}_{int,}}_{u,H,CYP27A1}=\frac{{CL}_{met,H,CYP27A1}}{\left(Liver\;Weight\times MPPGL\times CYP27A1\;abundance\right)\times (10^{6}/60)}$$

where ${fm}_{CYP27A1}$ is the fraction metabolized by the CYP27A1 pathway, ${{CL}_{int,}}_{u,H,CYP27A1}$ is the intrinsic clearance for the CYP27A1 pathway (µL/min/pmol CYP27A), and ${CL}_{met,H,CYP27A1}$ is the CYP27A1-specific whole liver clearance (L/h). Liver weight is 1737.11 g (simulation results of 1000 virtual healthy volunteers in the Simcyp Simulator), the microsomal protein per gram of liver (MPPGL) is 39.79 mg/g (simulation results of 1000 virtual healthy volunteers in the Simcyp Simulator), and the CYP27A1 abundance is 41.93 pmol/mg microsomal protein (Table S1).

Determination of fraction metabolized by CYP3A (${fm}_{CYP3A}$): Since there are limited experimental studies quantifying hepatic cholesterol, we first determined the fraction of cholesterol metabolized by CYP3A (${fm}_{CYP3A}$) based on a mathematical model using hepatic concentrations of cholesterol [22] and 4β-OHC [2]. The analysis yielded a ${fm}_{CYP3A}$ value of 2.1 × 10^−5^. We can assume that ${fm}_{CYP3A}$ is attributed mainly to CYP3A4 because CYP3A4 contributes to about 92% of cholesterol clearance by the CYP3A pathway [6]. Hence, the CYP3A4-specific hepatic CL_int_ (${CL}_{met,H,CYP3A4}$) and intrinsic clearance (${{CL}_{int,}}_{u,H,CYP3A4}$) were calculated as shown in Equations (3) and (4), respectively, using the CYP3A4 abundance (137 pmol/mg microsomal protein; a representative value for adult Caucasian healthy volunteers, used in the ‘Sim-Healthy Volunteer’ library default value). The rest of the total cholesterol clearance was attributed to additional HLM (${fm}_{HLM\;}$= 1- ${fm}_{CYP27A1}$ + ${fm}_{CYP3A}$) and the resulting HLM CL_int_ was calculated using Equations (5) and (6).

(5) $${CL}_{HLM}\;=\;{fm}_{HLM}\times {CL}_{met,H}$$

(6) $${CL}_{int,u,\;HLM}=\frac{{CL}_{HLM}}{\left(Liver\;Weight\times MPPGL\right)\times (10^{6}/60)}$$

where ${fm}_{HLM}\;\;$ is the fraction metabolized by additional HLM, ${CL}_{HLM}\;$ is the total hepatic clearance via the additional HLM pathway (L/h), and ${CL}_{int,u,\;HLM}$ is the intrinsic clearance for the additional HLM pathway (µL/min/mg).

The calculated intrinsic clearances were 7.46 × 10^−8^ µL/min/pmol CYP3A4, 3.13 × 10^−3^ µL/min/pmol CYP27A1, and 0.36 µL/min/mg HLM for CYP3A4, CYP27A1, and additional HLM, respectively. When simulations were run with these values, the steady-state 4β-OHC levels in healthy volunteers were significantly underpredicted. Also, the extent of DDI with rifampicin was underpredicted compared to the clinical study by Kasichayanula et al., 2014 [8]. Hence, the ${fm}_{CYP3A}$ required optimization.

Step 2: Optimization of Fraction Metabolized by CYP3A (${fm}_{CYP3A}$)

We manually optimized the CYP3A4 intrinsic clearance ${{CL}_{int,}}_{u,H,CYP3A4}\;\;$ such that the baseline and the rifampicin-induced 4β-OHC levels were recovered as per the Kasichayanula et al., 2014 study [8]. To account for contributions by the CYP3A5 and CYP3A7 pathways, we assumed the optimized CYP3A4 CL_int_ to be CYP3A clearance (${{CL}_{int,}}_{u,H,CYP3A})$ and their respective contributions to be 5.6% and 2.8%, according to in vitro experiments [4]. Finally, the CL_int_ for both CYP3A5 and CYP3A7 was calculated using Equation (7) as follows:

(7) $$\;{{CL}_{int,}}_{u,H,CYP3A5/7}={{CL}_{int,}}_{u,H,CYP3A}\times \frac{\%\;CYP3A5/7\;}{100}$$

where ${{CL}_{int,}}_{u,H,CYP3A5/7}$ is the intrinsic clearance for the CYP3A5/7 pathway (µL/min/pmol enzyme) and ${{CL}_{int,}}_{u,H,CYP3A}$ is the intrinsic clearance for the CYP3A pathway (µL/min/pmol enzyme) (combined CYP3A4, CYP3A5, and CYP3A7).

The respective ${fm,}_{CYP450}$ values were back-calculated according to Equation (8) as follows:

(8) $${fm,}_{CYP450}=\frac{{CL}_{met,H,CYP450}\;}{{CL}_{met,H}}$$

where ${fm,}_{CYP450}$ is the fraction metabolized by the CYP450 specific pathway, and $\;{CL}_{met,H,CYP450}$ is the CYP450-specific whole liver clearance (L/h).

A similar workflow was followed to determine intrinsic clearance values for 4β-OHC metabolism. The in vivo *t_1/2_* of 62 h reported by Bodin et al., 2002 [4], was used to calculate the ${CL}_{iv}$ (Equation (1)) and the total hepatic CL_int_ (Equation (2)).

The Bodin et al., 2002, study also reported that CYP27A1 contributes to 50% of 4β-OHC metabolism in vitro. Assuming an ${fm}_{CYP27A1}$ of 0.5, the ${CL}_{met,H,CYP27A1}$ and ${{CL}_{int,}}_{u,H,CYP27A1}$ were calculated using Equations (3) and (4). The remaining metabolism was attributed to additional HLM and ${CL}_{HLM}$, and ${CL}_{int,u,\;HLM}$ was calculated using Equations (4) and (5). The final ${{CL}_{int,}}_{u,H,CYP450}$ values for cholesterol and 4β-OHC metabolic pathways are shown in Table 1.

#### 2.2.3. Trial Design

Cholesterol was dosed daily at 1.73 mg/kg IV infusion for 24 h to achieve steady-state plasma cholesterol and 4β-OHC concentration according to a clinical study [2]. Since the ${fm}_{CYP3A}$ for cholesterol is very low, the simulations were run for at least 700 days. For the DDI studies, the perpetrator was administered after the 4β-OHC steady state was achieved.

### 2.3. PBPK Model Performance and Verification

Model performance was verified by comparing predicted and observed baseline plasma levels for cholesterol and 4β-OHC in default virtual ethnic populations in the Simcyp Simulator library, namely, North European Caucasian, North American White, North American Asian, North American African American, North American Hispanic-Latino, and Japanese. A virtual Korean population, first developed in the Simcyp simulator V19 (Certara UK Limited, Sheffield, UK) [29], was also used for the simulations. Next, the model was verified by comparing predicted and observed sex-specific baseline plasma levels of 4β-OHC. Next, we collected clinical DDI studies with rifampicin from the CDIS database and compared the elevated plasma 4β-OHC levels in six independent studies. The default CYP3A inducers and inhibitor files were used for DDI simulations, and the input parameters are described in Table S2. The trial design was set up such that the virtual population matched demographic characteristics such as age range and sex to comparator clinical trials. In each simulation, the trial design was matched to each clinical study as shown in Tables S3 and S4, and 10 virtual trials were generated. In clinical studies where cholesterol levels were reported, the infusion dose was adjusted such that the observed steady-state cholesterol levels were achieved, and thereafter the 4β-OHC levels were compared. Because there is uncertainty around the values of CYP27A1 abundance and cholesterol infusion dose, we performed a local sensitivity analysis to assess their impact on cholesterol and 4β-OHC plasma concentrations.

### 2.4. Statistical Assessment of Model Prediction Accuracy

The simulated vs. observed steady-state plasma concentration ratio was used to assess model performance together with visual predictive checks. Model fit was assessed using average fold error (AFE) and absolute average fold error (AAFE), calculated as follows:

(9) $$AFE=10^{\frac{1}{n}\times \sum {log}_{10}\left(\left|\frac{{Predicted}_{i}}{{Observed}_{i}}\right|\right)}$$

(10) $$AAFE=10^{\frac{1}{n}\times \sum \left|\log_{10}\left(\frac{{Predicted}_{i}}{{Observed}_{i}}\right)\right|}$$

where ${Prediced}_{i}$ and ${Observed}_{i}$ are the predicted and observed steady-state 4β-OHC concentration in the study i, and n is the number of studies. The predicted/observed ratio indicates the predictive accuracy of plasma concentration in each study and having ratios lower than 2 is a commonly accepted model assessment criterion [30,31]. The AFE indicates the bias of the prediction model and equally weights over- and under-prediction across multiple studies (i.e., AFE > 1 indicates overprediction and AFE < 1 indicates underprediction). AAFE quantifies absolute differences from the observed values. If the AFE value is close to 1 and AAFE is below 2; the simulation results are usually considered acceptable [32]. Finally, the predictive performance of the model for DDI studies was assessed with the Guest criterion [33].

### 2.5. PBPK Model Application

The verified PBPK model was subsequently applied to predict changes in 4β-OHC levels in specific populations and following co-administration with other CYP3A inducers.

#### 2.5.1. CYP3A4 Polymorphism

CYP3A4 is a highly polymorphic enzyme with more than 35 variants reported to date. Most variants appear at very low frequency in the population and do not alter the enzymatic activity. However, a single nucleotide polymorphism (CYP3A4*22) was associated with a 1.7- and 2.5-fold lower expression and activity, respectively, compared to the wild type (CYP3A4*1) [34]. To investigate whether the CYP3A4*22 variant is of clinical importance in modulating 4β-OHC levels, we first analyzed the literature to determine the population frequency of the CYP3A4*22 allele and the change to the CYP3A4 enzyme abundance. Based on our analysis, we created three virtual populations corresponding to wild-type (CYP3A4*1/*1), heterozygous carriers (CYP3A4*1/*22), and homozygous carriers (CYP3A4*22/*22) using the virtual North European Caucasian population as a reference. Simulations were run with 20 subjects, 10 trials, a 20–50-year age range, and 50% females. The cholesterol and 4β-OHC levels were then compared to the observed clinical data.

#### 2.5.2. CYP3A5 Polymorphism

Like CYP3A4, CYP3A5 is a highly polymorphic enzyme with two dominant variants, namely, CYP3A5*1 and CYP3A5*3. The effect of CYP3A5 polymorphisms on the clearance of CYP3A substrates is well studied. Individuals with homozygous CYP3A5*1/*1 show normal (wild type) clearance of CYP3A substrates (termed extensive metabolizers (EMs), while CYP3A5*1/*3 and CYP3A5*3/*3 individuals show reduced clearance of CYP3A substrates, termed intermediate (IMs) and poor metabolizers (PMs), respectively. Unlike CYP3A4, CYP3A5 polymorphisms are observed at a higher frequency in certain populations. For example, populations of Asian and African descent exhibit a higher frequency of CYP3A5 EMs, while the North European Caucasian population exhibits higher CYP3A5 PMs [35]. To investigate whether individuals with distinct CYP3A5 polymorphisms show varied 4β-OHC levels, we developed CYP3A5 EM and PM variants of Asian and African/American populations and compared the 4β-OHC levels. The trial design involved 20 trials, 10 individuals, 20–50 years, and 50% females.

#### 2.5.3. RA Patients

Several clinical studies have shown suppressed CYP3A4 expression and activity in inflammatory conditions, such as RA [36], mainly due to downregulation by cytokines such as interleukin-6 (IL-6) and tumor necrosis factor alpha (TNFα) [37]. As a result, the CYP3A4 substrate alprazolam has reduced clearance in inflammatory conditions [38]. In a previous study, plasma 4β-OHC levels were found to be lower in RA patients compared to healthy individuals [39]. Hence, we used the default RA population within the Simcyp Simulator to simulate changes in 4β-OHC and compared them to healthy volunteer data. Simulations were performed with 30 individuals in 10 trials (300 subjects), 19–76 years, 80% females.

#### 2.5.4. Effect of Other CYP3A4 Inducers

The verified PBPK model was subsequently applied to predict changes in plasma 4β-OHC levels following co-administration with moderate CYP3A inducers, namely, carbamazepine, phenytoin, efavirenz, and phenobarbital. Simulations were performed with 10 virtual trials of 20 healthy participants, 20–50 years, and 50% female. Inducers were co-administered after 700 days of cholesterol infusion at a dosage regimen of carbamazepine 600 mg q.d., efavirenz 600 mg q.d., and phenytoin 300 mg q.d. for 400 days to mimic long-term therapy. The default Simcyp Simulator compound library files for SV-Carbamazepine (including SV-Carbamazepine-10,11-epoxide), SV-Phenytoin, SV-Efavirenz, and SV-Phenobarbital were used in the simulations (Table S2). Finally, we also simulated a DDI study with rifampicin 600 mg q.d., 30 days in healthy volunteers and in the RA population and used the default SV-Rifampicin-MD file.

### 2.6. Statistical Analysis of 4β-OHC Levels in Different Groups

All the reported data is in mean ± S.D. unless otherwise stated. The 4β-OHC levels in different groups were compared using either ordinary one-way analysis of variance (ANOVA) with Tukey’s multiple comparisons test or the unpaired *t*-test with Welch’s correction. Statistical analyses were performed using GraphPad Prism 10.1.1 (GraphPad Software, Boston, MA, USA). *p*-values < 0.05 (*) were considered statistically significant.

## 3. Results

### 3.1. Ethnic and Sex-Specific Differences in Baseline 4β-OHC Levels

Based on our inclusion/exclusion criteria, we collected data for plasma concentration of 4β-OHC from 38 studies. The mean ± SD steady-state plasma concentration across all studies in healthy individuals was 29.85 ± 14.87 ng/mL. Similarly, we collected data for cholesterol plasma concentration from three independent studies, and the mean ± SD value was 1.74 ± 0.26 mg/mL.

**Figure 3:**
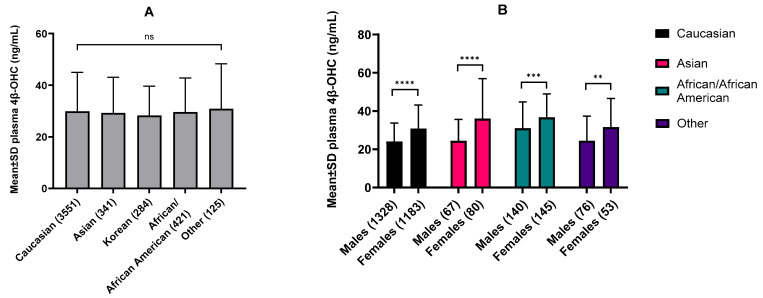
(**A**) Baseline plasma 4β-OHC levels in different ethnicities expressed as mean ± S.D. (**B**) and sex-specific 4β-OHC levels within ethnicities. Data was collated for meta-analysis with the number of total individuals analyzed mentioned in the parentheses. Our analysis showed negligible statistical difference in baseline 4β-OHC levels between ethnicities but consistently higher levels in females compared to males within each ethnicity. (**A**) Different ethnicities were compared using the ordinary one-way ANOVA with Tukey’s multiple comparison test. (**B**) Males and females within ethnicities were compared using an unpaired *t*-test with Welch’s correction. ** *p* < 0.01, *** *p* < 0.001, **** *p* < 0.0001, ns = not significant.

Our meta-analysis comprised data for 4466 individuals and revealed a negligible statistical difference in mean plasma 4β-OHC levels among Caucasian (28 studies), Asians (10 studies), Koreans (6 studies), African Americans (4 studies), and other populations (4 studies) (Figure 3A). Females exhibited an average 31% higher plasma 4β-OHC levels than males on average. Asian females exhibited the highest (47%) difference, while African American females showed the lowest (18%) difference in 4β-OHC levels compared to their male counterparts (Figure 3B).

### 3.2. PBPK Model Development and Performance Verification

**Figure 4:**
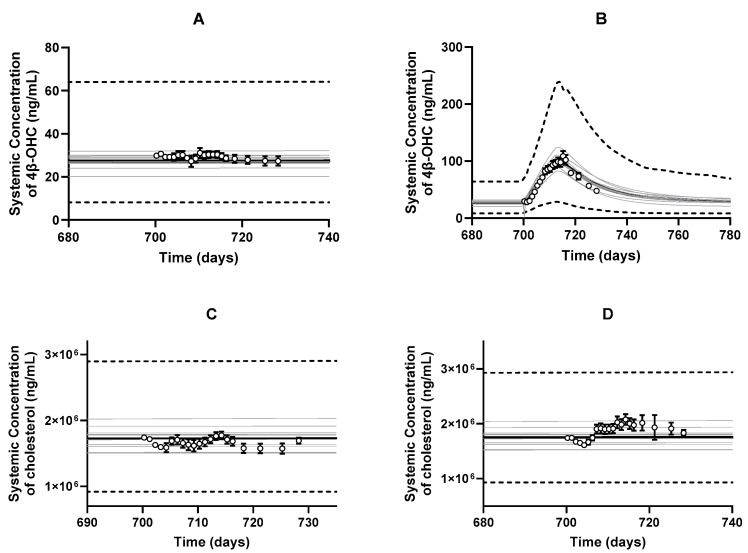
Predicted and observed (open circles) arithmetic mean plasma concentration–time profiles of (**A**) baseline 4β-OHC, (**B**) rifampicin-induced 4β-OHC, (**C**) baseline cholesterol, and (**D**) rifampicin-driven cholesterol levels. Simulations were performed with the following trial characteristics: 10 trials × 12 subjects, 20–50 years, a proportion of 5% females, multiple populations (North American Caucasian: North American African American: North American Asian; 0.56:0.41:0.03) with orally administered multiple doses of 600 mg q.d. rifampicin for 13 days [8]. The black lines represent the population mean plasma concentration–time profiles, the grey lines represent the predictions from individual trials, whereas the dashed lines represent the 5th and 95th percentiles of all simulated subjects’ values. Observed data is presented as mean ± S.D. values according to the Kasichayanula et al. study in 2014 [8].

One of the critical steps in the model development was to optimize the ${fm}_{CYP3A}$ and calculate the ${{CL}_{int,}}_{u,H,CYP3A}$. The optimized ${fm}_{CYP3A}$ values for CYP3A4, CYP3A5, and CYP3A7 for cholesterol metabolism were 1.17 × 10^−4^, 7.17 × 10^−6^, 3.59 × 10^−6^, respectively. When simulations were run with the optimized intrinsic clearance values, we recovered the baseline 4β-OHC and cholesterol levels as per the study by Kasichayanula et al. [8] (Figure 4A,C). Importantly, we recovered the induced 4β-OHC level in response to multiple dose co-administration of rifampicin (Figure 4B). When the DDI with multiple dose ketoconazole was simulated, predictions were slightly overpredicted but fell within the two-fold range (0.62-fold) (Figure S1A). Interestingly, the predicted cholesterol levels were not impacted either due to rifampicin or ketoconazole treatments (Figure 4D and Figure S1B). The observation is not unexpected as the plasma cholesterol concentration is orders of magnitude higher than 4β-OHC, and the ${fm}_{CYP3A}$ of cholesterol is quite small; therefore, minor changes in CYP3A activity may not be significantly reflected in cholesterol levels.

**Figure 5:**
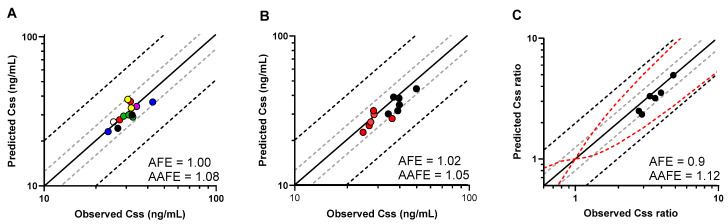
(**A**) Predicted and observed (filled circles) mean plasma baseline 4β-OHC levels (C_ss_) in different virtual populations depicted in different colours as follows: red—North American White; green—North European Caucasian; yellow—North American African American; violet—North American Asian; white—North American Latino; black—Korean; blue—Japanese. (**B**) Predicted and observed mean plasma baseline 4β-OHC levels (C_ss_) in males (red circles) and females (black circles). (**C**) Predicted and observed mean plasma concentration ratio (C_ss_ ratio) of rifampicin-induced to baseline 4β-OHC levels (black circles) examined in six DDI studies. C_ss_ and C_ss_ ratios are shown with the solid line representing the Line of Unity, grey dashed lines indicating 1.25-fold, and black dashed lines showing a 2-fold deviation from the respective observed value; the red dashed lines mark the prediction success limits proposed by Guest et al. [33].

To verify the model, we designed simulations to replicate specific clinical studies where 4β-OHC levels were reported (Table S3). As shown in Figure 5A, the model predictions for 4β-OHC baseline levels in different ethnic populations were within 1.25-fold of the observed values (AFE = 1; AAFE = 1.08). Additionally, sex-specific differences between males and females within the ethnicities were also well captured (AFE = 1.02; AAFE = 1.05) (Figure 5B and Table S3). Next, we simulated other DDI studies with multiple dose treatment of rifampicin, and the model predicted the induced 4β-OHC levels within less than the 1.25-fold error of the observed values from six independent studies (AFE = 0.9; AAFE = 1.12) (Figure 5C and Table S4).

**Figure 6:**
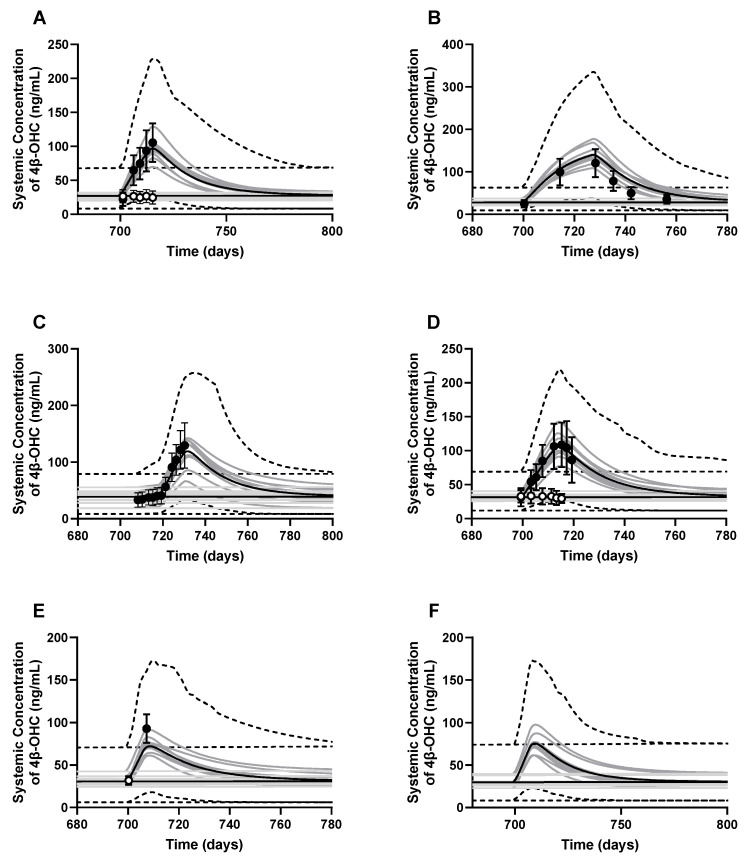
Simulated and observed baseline 4β-OHC plasma concentration–time profiles (white dots) and rifampicin-induced 4β-OHC levels (black dots). The DDI studies simulated were (**A**) Dutreix et al., 2014 [41]; (**B**) Stoch et al., 2016 [42]; (**C**) Wiesinger et al., 2020 [43]; (**D**) Einholf et al., 2017 [41,44]; (**E**) Marschall et al., 2005 [45]; and (**F**) a baricitinib-rifampicin DDI [46]. Trial design for each simulation is described in Table S4. The dark lines represent the mean plasma concentration–time profiles, the grey lines represent the predictions from individual trials, whereas the dashed lines represent the 5th and 95th percentiles of all simulated subjects’ values.

Additionally, the model predicted a mean 4β-OHC elimination half-life post rifampicin administration of 23.81 ± 5.32 days, which slightly overpredicts the reported values of 10–17 days from clinical studies or mathematical calculations [5,40]. Plasma concentration-time profiles for the baseline and induced 4β-OHC in six DDI studies are shown in Figure 6. Lastly, local sensitivity analysis on CYP27A1 abundance in healthy volunteers showed that a decrease in CYP27A1 abundance from 40 to 20 pmol/mg microsomal protein increased cholesterol (Figure S2A) and 4β-OHC (Figure S2B) plasma levels by 1.16- and 1.56-fold, respectively. Similarly, an increase in cholesterol infusion dose from 1 to 3 mg/kg led to an increase in cholesterol levels from 0.97–2.93 mg/mL (Figure S2C) and 4β-OHC levels from 15 to 45 ng/mL (Figure S2D).

### 3.3. PBPK Model Applications

#### 3.3.1. CYP3A4*22 Carriers Show Reduced CYP3A4 Protein Content and Baseline 4β-OHC Levels

**Figure 7:**
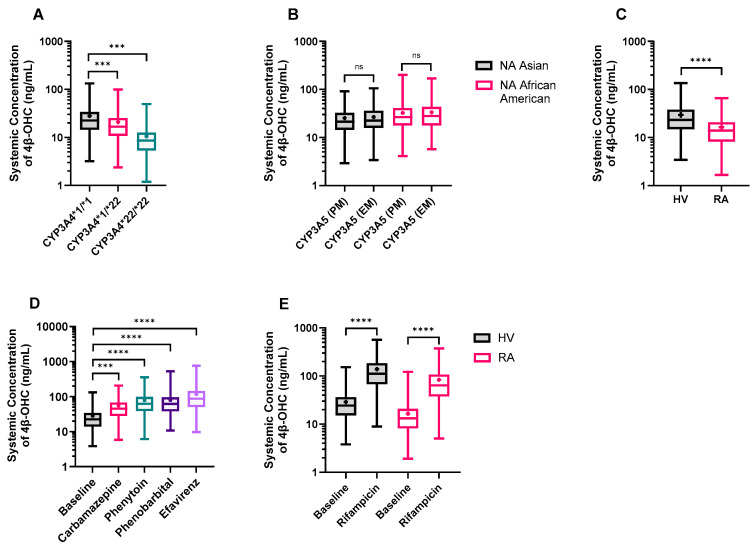
Model application: Predicted plasma 4β-OHC concentrations in (**A**) CYP3A4 polymorphic populations; (**B**) CYP3A5 polymorphic populations in North American (NA) Asian and African American populations; (**C**) healthy volunteer (HV), and rheumatoid arthritis (RA) populations; (**D**) the absence and presence of moderate CYP3A inducers in HV, and (**E**) the absence and presence of rifampicin in RA patients. The trial characteristics and the reference ethnic population are mentioned in Tables S5 and S6. Data is presented as a box and whisker plot showing minimum, lower quartile, median, upper quartile, and maximum 4β-OHC values. The length of the box refers to the interquartile range, and the whiskers end at the minimum and maximum values. The ‘+’ shows the mean 4β-OHC value. Different groups were compared using the ordinary one-way ANOVA with Tukey’s multiple comparison test (**A**,**B**,**D**,**E**) or unpaired *t*-test with Welch’s correction (**C**). *** *p* < 0.001, **** *p* < 0.0001, ns = not significant.

Our meta-analysis showed that the CYP3A4*1/*22 and CYP3A4*22/*22 frequencies in the Caucasian population are only 1.1% and 0.04%, respectively, while these polymorphisms were not observed in Asian or African American populations. Due to extremely low frequency, separate CYP3A4*1/*22 and CYP3A4*22/*22 only virtual populations were developed, and 4β-OHC levels were simulated. We found one study where the effect of CYP3A4*22 genotype on CYP3A4 abundance was measured in HLM in four Caucasian individuals. The geometric mean of CYP3A4 content was 49.7 pmol/mg microsomal protein for the wild-type (n = 19) and 8.7 pmol/mg microsomal protein for heterozygous CYP3A4*22 carriers (n = 4), leading to a protein content ratio of 0.175 [47]. However, with these values, there was an underprediction in the clearance of CYP3A4 substrates such as midazolam. Due to the limited number of samples carrying the CYP3A4*22 allele (n = 4), the clinical data for midazolam was used to estimate the difference in hepatic CYP3A4 abundance between different CYP3A4*22 genotypes (i.e., hetero- and homo-zygotic carriers) using a reverse translational approach. In the first step, the in vitro ${{CL}_{int,}}_{u,H,CYP3A4}$ and ${{CL}_{int,}}_{u,H,CYP3A5}$ of midazolam was added and scaled to ${CL}_{met,H,CYP3A}$ using mean microsomal protein per gram of liver (MPPGL) and liver weight. Next, the well-stirred liver model was used to calculate the ${CL}_{met,H}$ of midazolam from ${CL}_{met,H,CYP3A}$, hepatic blood flow and fraction unbound in blood. For CYP3A4*1/*22 and CYP3A4*22/*22 individuals, the ${CL}_{met,H}$ was lowered by 20% and 50%, respectively, based on plasma concentrations of midazolam [48] and tacrolimus [49]. Finally, the ${{CL}_{int,}}_{u,H,CYP3A4}$ was back calculated from reduced ${CL}_{met,H}$ and converted from L/h to μL/min/mg microsomal protein using the MPPGL and liver weight. This resulted in CYP3A4 abundance of 100.86 pmol/mg microsomal protein in CYP3A4*1/*22 carriers and 51.27 pmol/mg microsomal protein in CYP3A4*22/*22 carriers. The default CV of 41% for CYP3A4 EMs was assumed to be the same for all genotypes. Importantly, it was assumed that the CYP3A4*22 allele only resulted in altered CYP3A4 abundance while other scaling factors such as enzyme activity and MPPGL were unaffected. To verify the calculated CYP3A4 abundances, a simulation was run for 12 heterozygous carriers of CYP3A4 (10 trials × 12 individuals, 45–65 years, 38.4% women) to replicate the Elens et al., 2013 [50], study. The simulation using the retrograde calculated protein abundance for CYP3A4*1/*22 gave a realistic estimate of the clinically observed data for midazolam pharmacokinetics in CYP3A4*1/*22 individuals [50] (Figure S3). Once the CYP3A4 abundances in polymorphic populations were verified, we simulated 4β-OHC levels in three virtual populations, namely, CYP3A4*1/*1 (EM), CYP3A4*1/*22 (IM), and CYP3A4*22/*22 (PM). As expected, the predicted 4β-OHC levels were significantly reduced to 0.74-fold in IM and 0.38-fold in PM populations in line with the observed value from clinical studies (Figure 7A and Table S5).

#### 3.3.2. The Impact of CYP3A5 Polymorphisms

Next, we simulated the 4β-OHC levels in CYP3A5 EM and PM only populations separately. Our simulations revealed that CYP3A5 EMs had marginally higher, but not statistically significant 4β-OHC levels compared to CYP3A5 PMs in both North American Asian and North American African American populations. The EM/PM ratio for predicted 4β-OHC levels was 1.07 and 1.04 for North American Asian and North American African American populations, respectively (Figure 7B and Table S5), in line with the observed data of 1.13 and 1.07 [5,51,52], respectively. The marginal differences in baseline could be attributed to the minor role played by CYP3A5 (and CYP3A7) in overall 4β-OHC formation.

#### 3.3.3. Rheumatoid Arthritis (RA) Patients Exhibit Lower 4β-OHC Levels

An RA virtual population was developed in the Simcyp Simulator with physiological data collected from the literature [53]. Simulations of 1000 virtual individuals of healthy and RA populations (20–65 years and 50% females) revealed that cardiac output was 5% higher while hematocrit values were 17% lower in RA patients compared to healthy volunteers. More importantly, the mean CYP3A4 abundance in RA patients (82.07 pmol/mg microsomal protein) was 40% lower than that in healthy individuals (138.89 pmol/mg microsomal protein). When simulations were performed in the RA population, the predicted mean 4β-OHC levels were 0.55-fold lower than in healthy individuals (Figure 7C and Table S5), thus reflecting the reduced CYP3A4 abundance in RA patients.

#### 3.3.4. DDI Simulation with Other CYP3A4 Inducers

The model was applied to predict changes in 4β-OHC levels with co-administration of CYP3A inducers such as carbamazepine, phenytoin, phenobarbital, and efavirenz. The simulations were conducted in healthy volunteers with the same age range, proportion of females, and default dose for 400 days to minimize the effects of covariates and to mimic long-term therapy. Co-administration of carbamazepine, phenytoin, phenobarbital, and efavirenz caused 2.22-, 2.76-, 2.81-, and 4.15-fold increases in 4β-OHC levels, respectively, in line with their CYP3A inductive effects (Figure 7D and Table S6). Finally, the simulated DDI with rifampicin in the RA population (5.02-fold) was comparable to a DDI simulated in healthy volunteers (4.76-fold) (Figure 7E and Table S6).

## 4. Discussion

The identification and use of endogenous biomarkers in clinical DDI studies is a subject of active research. For example, coproporphyrin I (CP-I), a by-product of heme synthesis, is being explored as an endogenous biomarker for organic anion transporting polypeptides 1B1/1B3 (OATP1B) transporter activity in clinical studies [54] and PBPK models [55]. As for 4β-OHC, its utility as a CYP3A-specific biomarker for clinical DDIs is highlighted, as several novel drugs such as enasidenib [11], ivosidenib [56], PF-06282999 [57], and PF-05251749 [58] were investigated for their CYP3A inductive effect on 4β-OHC. However, there has been limited progress in developing mechanistic models to describe 4β-OHC kinetics. To our knowledge, this is the first PBPK model that accurately predicted the baseline 4β-OHC levels in healthy adults of different ethnicities, explored sex-specific differences in 4β-OHC levels, and recapitulated the impact of co-administered CYP3A inducers on the plasma levels of 4β-OHC.

To develop the Simcyp Simulator cholesterol and 4β-OHC files, a meta-analysis of the available literature was conducted to establish baseline plasma levels in different ethnicities. Our meta-analysis revealed minimal difference in mean 4β-OHC plasma levels between ethnicities but significantly higher baseline levels in females within an ethnic population. This is likely the result of the sensitivity of the 4β-OHC plasma levels to the overall hepatic abundance of CYP3A. To assess this impact, we simulated 1000 virtual individuals of North European Caucasian and North American Asian populations (20–50 years and 50% females). The 16% lower CYP3A4 hepatic abundance in North American Asians compared to North European Caucasians was compensated by a higher proportion of CYP3A5 extensive metabolizers (48% in Asians versus 17% in Caucasians), leading to an 115% higher CYP3A5 expression in North American Asians. Therefore, the difference in baseline 4β-OHC levels between the two populations was not statistically significant. Females displayed higher 4β-OHC plasma levels than males [59], which was statistically significant due to females exhibiting a 24% higher hepatic CYP3A4 abundance than males in 1000 simulated healthy volunteer individuals. This indicates that overall sex is an important covariate for achieving the appropriate 4β-OHC plasma levels in virtual populations.

Once the baseline values were established from meta-analysis, we developed a PBPK model of cholesterol and 4β-OHC using a middle-out approach. One of the first steps was to determine the ${fm}_{CYP3A}\;$ of cholesterol. In our literature search, we came across only one in vitro study investigating the metabolism of cholesterol and 4β-OHC by different CYP450 pathways [4]. The in vitro assays in this study were performed where cholesterol or 4β-OHC were incubated with recombinant enzymes separately and the percentage of substrate metabolized was measured. In the absence of other data, these values were used to guide the ${fm}_{CYP450}\;$ calculations. However, these assay results do not reflect the true in vivo ${fm}_{CYP3A}$ of cholesterol, and hence, the in vivo ${fm}_{CYP3A}\;$ values were optimized using a DDI study with rifampicin [8].

In this model, we incorporated the hepatic abundance of CYP27A1, which was derived from a single proteomics study [26], and therefore assumed it to be the same for different populations. However, like CYP3A, CYP27A1 abundance could be expected to vary across ethnicities, and the sensitivity analysis on CYP27A1 abundance reflects its significant effect on 4β-OHC levels (Figure S2A,B). Hence, the accurate CYP27A1 abundance from various sources and its associated variability needs to be accounted for in the future. On the other hand, due to lack of hepatic abundance data, the metabolism via the CYP7A1 pathway was attributed to an additional HLM clearance to achieve the steady-state plasma 4β-OHC levels. As a result, any perturbation of CYP7A1 that may affect the 4β-OHC levels could not be captured in this model. This could be important since there is evidence to show that rifampicin significantly suppressed CYP7A1 mRNA expression in hepatocytes in vitro [60,61]. Therefore, rifampicin-induced CYP7A1 suppression may lead to higher 4β-OHC levels, which may confound its sole CYP3A inductive effects. Since we optimized the ${fm}_{CYP3A}\;$ and the ${CL}_{int,\;CYP3A}\;$ to a clinical DDI study with rifampicin; we might need to consider ${fm}_{CYP7A1}$ and reevaluate the ${fm}_{CYP3A}$ provided there is robust clinical data on the contribution of rifampicin-driven CYP7A1 suppression on 4β-OHC levels. Nevertheless, the optimized ${fm}_{CYP3A}\;$ and the ${CL}_{int,\;CYP3A}$ used in the model predicted the baseline 4β-OHC levels in different ethnic populations within 1.25-fold error of observed data with minimal bias and high precision. We also accurately predicted the sex-specific differences in baseline 4β-OHC levels, thus providing confidence in the optimized ${fm}_{CYP3A}\;$ and the ${CL}_{int,\;CYP3A}$ values.

Once the mean baseline 4β-OHC levels were well captured, the model was verified with six independent DDI studies with 600 mg q.d. multiple dose rifampicin. We used the default rifampicin multiple dose file from the Simcyp Simulator library, where the CYP3A4 and CYP3A5 induction parameters, namely, maximal fold induction (Ind_max_ = 16) and concentration at half-maximal fold induction (IndC_50_ = 0.32 μM), were previously optimized using clinical data [62]. These default induction parameters predicted that the elevated 4β-OHC levels were in good agreement with the observed clinical data (Figure 6 and Table S4). Finally, having verified the capacity of the model to recover the potent CYP3A induction with rifampicin, the model was extended to predict DDIs with four moderate CYP3A4 inducers. Amongst the four CYP3A4 inducers investigated, efavirenz showed the highest (4.15-fold) induction of 4β-OHC levels compared to the lowest (2.22-fold) induction by carbamazepine (Figure 7D, Table S6). This observation is in line with their relative induction potencies estimated by their Ind_max_/IndC_50_ ratios, that is, 2.60 for efavirenz [63] and 0.16 for carbamazepine [64]. However, when the CYP3A4 inhibition study was simulated with ketoconazole, the model slightly overpredicted the reduced plasma 4β-OHC levels (Figure S1). This contrasts with observed clinical studies where the reduction in plasma levels is not significant [8]. As a result, appropriate caution must be exercised when using the model to predict the change in 4β-OHC levels due to CYP3A4 inhibition, as it might lead to overprediction of the DDI. The overprediction in plasma 4β-OHC levels could be due to higher ${fm}_{CYP3A}$ values, which were optimized to recover the induction DDI with rifampicin [8]. Considering the CYP7A1 suppressive effects of rifampicin, the higher plasma 4β-OHC levels could overpredict the true ${fm}_{CYP3A}\;$ values. Hence, an understanding of the contribution of in vivo CYP7A1 suppression in elevating 4β-OHC levels could better guide us in estimating the true and possibly lower in vivo ${fm}_{CYP3A}\;$ for 4β-OHC elimination and reproduce the DDI with strong CYP3A inhibitors.

One of the limitations of the model is that the predicted variability demonstrated by the 5th and 95th percentiles (Figure 4 and Figure 6) in cholesterol and 4β-OHC levels was much higher than observed clinical variability. Prior studies have shown that perturbation of cholesterol and 4β-OHC levels triggers CYP7A1-mediated bile acid regulatory mechanism independent of the CYP3A pathway [65,66]. For example, at higher cholesterol levels, bile acid synthesis is upregulated (CYP7A1 pathway), while 4β-OHC levels (CYP3A pathway) might remain unchanged. Such complex homeostatic regulation of cholesterol and 4β-OHC cannot be considered in the current model, and hence, it is challenging to capture the resulting clinically observed variability. However, conservatively, it is more acceptable to have over-predicted variability than under-predicted variability, as one could more readily pinpoint at risk outliers.

One of the advantages of PBPK modelling is to investigate the effect of different scenarios, such as disease and CYP450 polymorphisms, on pharmacokinetic parameters. In this study, we explored the effects of CYP3A4*22 and CYP3A5*1 and CYP3A5*3 polymorphisms on 4β-OHC levels. Prior clinical studies have shown that CYP3A4*22 carriers have reduced CYP3A4 substrate clearance and increased systemic exposure. For example, tacrolimus and quetiapine exposure increased by 1.5- and 1.88-fold, respectively, in CYP3A4*1*22 compared to CYP3A4*1/*1 individuals [49,67,68]. Based on the clinical studies, we calculated the reduced CYP3A4 abundance in the CYP3A4*22 polymorphic populations and simulated the baseline 4β-OHC levels. The reduced CYP3A4 abundance translated well to the reduced predicted 4β-OHC levels in CYP3A4*1/*22 carriers and CYP3A4*22/*22 carriers (Figure 7A, Table S5) in line with published reports [69,70]. Like CYP3A4 polymorphisms, we also investigated the effects of CYP3A5 polymorphisms on 4β-OHC levels. CYP3A5*3 carriers are highly expressed in the Asian and African American populations. For example, our meta-analysis revealed that CYP3A5*1/*3 and CYP3A5*3/*3 carriers are about 51.8% and 48.2% of the total Asian population and 27.5% and 72.5% of the total African American population (default Simcyp Simulator values derived from [35]). In terms of substrate clearance, it is well established that CYP3A5*1 carriers show higher substrate clearance and lower systemic exposure. For example, in renal impairment patients, the CYP3A5*1 carriers displayed about 2.4-fold higher tacrolimus clearance compared to CYP3A5*3 carriers [71]. The model predicted the higher 4β-OHC levels in the CYP3A5 EM population compared to PM in line with observed clinical data [5,51,52]. Overall, the simulations in CYP3A4 and CYP3A5 polymorphic populations further validated the optimized ${fm}_{CYP3A}\;$ and ${CL}_{int,\;CYP3A}$ values.

Next, the impact of disease on 4β-OHC levels was investigated in a virtual RA population. The reduced CYP3A4 abundance due to suppressive effects of proinflammatory IL-6 and TNFα cytokines in RA was well reflected in 0.6-fold lower predicted baseline and induced 4β-OHC levels in RA patients compared to healthy individuals. However, reduced CYP3A4 abundance and therefore reduced cholesterol clearance did not significantly increase the predicted cholesterol levels; possibly compensated by other cholesterol metabolic pathways such as CYP3A5. Since the baseline cholesterol levels might be affected due to disease [72] and immunosuppressive therapy [73], we performed a sensitivity analysis on the cholesterol infusion dose to assess its impact on 4β-OHC levels (Figure S2C,D). A gradual increase in the cholesterol infusion dose leading to an increase in 4β-OHC levels suggested that the measurement of baseline cholesterol levels in the disease population is crucial to establish a baseline for 4β-OHC levels. Like the studies for the CYP3A4*22 allele, there is scant data on the impact of RA on 4β-OHC levels. We sourced one study where 4β-OHC levels were measured in RA patients. Wollmann et al., 2016, reported that the median 4β-OHC levels were reduced by 0.76-fold in RA compared to healthy individuals [39]. Additionally, in patients with RA, there was a 10-fold variability in 4β-OHC levels, which largely overlapped with the noninflammatory reference subjects. This indicates that the model predicted the drop in 4β-OHC levels in line with this reported study, thus underlining our optimized ${fm}_{CYP3A}$ and ${CL}_{int,\;CYP3A}$ values. The reduced CYP3A4 abundance and therefore reduced clearance of CYP3A4 substrates in RA patients increases the potential risk of adverse drug reactions. Using this model, we can identify individuals exhibiting the highest CYP3A4 suppression, namely, the greatest drop in 4β-OHC levels, to optimize, if necessary, the dose of CYP3A4 substrates to avoid adverse effects.

One of the inherent limitations of the 4β-OHC biomarker is that it mainly reflects hepatic rather than intestinal CYP3A activity [3]. This is not unexpected, as the liver is the main site of 4β-OHC synthesis and metabolism [24]. Hence, when evaluating CYP3A-mediated DDIs, it is necessary to evaluate if lack of intestinal CYP3A perturbation and its minimal effect on 4β-OHC levels might underestimate the DDI between novel CYP3A substrates and inhibitors/inducers. For example, in a previous study, Chen et al., 2022, reported that carbamazepine exhibited stronger CYP3A4 induction in the gut compared to the liver, while the efavirenz showed predominantly higher hepatic CYP3A induction [74]. As a result, the relatively minor hepatic CYP3A induction by carbamazepine along with lower induction potency could contribute to the lower induced 4β-OHC levels compared to efavirenz.

## 5. Conclusions

In conclusion, the PBPK model we developed for cholesterol and 4β-OHC accurately predicted the baseline and induced 4β-OHC levels in good concordance with clinical studies. This verified model can be applied to accompany CYP3A induction studies before or in parallel to a clinical trial setting. It can also be used to evaluate already existing clinical trials, as shown as part of the verification. As cholesterol and 4β-OHC share the same metabolic pathways, more research is needed to also address the elimination of the metabolite more mechanistically. As our fit-for-purpose model for CYP3A hepatic induction evaluation can be easily extended, it is in fact a model that is framed already for an even more mechanistic future use.

## Figures and Tables

**Table 1:** Input parameters used for the cholesterol and 4β-OHC PBPK parts of the PBPK model.

	Cholesterol		4β-OHC	
Parameter	Value	Source/Reference	Value	Source/Reference
Physicochemical Parameters				
Molecular weight (g/mol)	386.65	HMDB ID (HMDB0000067)	402.35	HMDB (HMDB0013643)
log P	7.02	HMDB ID (HMDB0000067)	6.16	HMDB ID (HMDB0013643)
Compound type	Neutral		Neutral	
Blood Binding Parameters				
B/P	0.55	[18]	0.55	[18]
fu_p_	0.0021 Predicted (QSAR)	[20]	0.0051 Predicted (QSAR)	[20]
Reference binding component	Apolipoprotein B	[21]	Apolipoprotein B	[21]
Protein reference concentration (µM)	620	Optimized. Please see the methods Section 2.2 for details.	620	Optimized. Please see the methods Section 2.2 for details.
Distribution	Minimal PBPK Model			
*V_ss_* (L/kg)	0.10	User input	0.05	User input
Liver K_p_	3.07	Calculated from [23]. See Table S1.	3.87	Calculated from [23]. See Table S1.
Elimination	Enzyme Kinetics			
CYP3A4 CL_int_ (µL/min/pmol)	4.17 × 10^−7^	Optimized. Please see the methods Section 2.2.2 for details.	N.A.	Optimized. Please see the methods Section 2.2.2 for details.
CYP3A5 CL_int_ (µL/min/pmol)	2.55 × 10^−8^		N.A.	
CYP3A7 CL_int_ (µL/min/pmol)	1.27 × 10^−8^		N.A.	
CYP27A1 CL_int_ (µL/min/pmol)	3.13 × 10^−3^		0.02	
Additional HLM CL_int_ (μL/min/mg protein)	0.350		0.84	

N.A.: not applicable; fu_p_: fraction unbound in plasma; B/P: blood-to-plasma ratio; HMDB: Human Metabolome Database; V_ss_: volume of distribution at steady state; K_p_: tissue-to-plasma partition ratio; CL_int_: in vitro intrinsic clearance; QSAR: quantitative structure-activity relationship.

## Data Availability

The original contributions presented in the study are included in the article/Supplementary Materials, further inquiries can be directed to the corresponding authors.

## Supplementary Materials

The following supporting information can be downloaded at: https://www.mdpi.com/article/10.3390/pharmaceutics16101284/s1, Table S1. Calculation of liver Kp of cholesterol and 4β-OHC. Table S2. Final input parameters for CYP3A inducers and inhibitors. Table S3. Trial design, population characteristics of clinical studies used to verify the predictions of baseline 4β-OHC plasma concentrations in different ethnicities and sexes. Table S4. Trial design, predicted and observed 4β-OHC levels of DDI simulations for the cholesterol and 4β-OHC model development and verification. Table S5. Model application: Predicted plasma 4β-OHC concentrations in CYP3A4 and CYP3A5 polymorphic populations, RA and HV populations. Table S6. Model application: Predicted plasma 4β-OHC concentrations in the absence and presence of moderate CYP3A inducers in healthy volunteers and RA patients. Figure S1. Predicted and observed (open circles) arithmetic mean plasma concentration–time profiles of (A) ketoconazole-driven cholesterol and (B) ketoconazole-driven 4β-OHC levels. Figure S2. Automated sensitivity analysis was performed to investigate the impact of CYP27A1 abundance and cholesterol dose on (A, C) the predicted cholesterol plasma concentration and (B, D) the predicted 4β-OHC plasma concentration. Figure S3. Verification of reduced CYP3A4 abundance in the CYP3A4*1/*22 population with an independent midazolam IV study.
