# Supplementary material for: Applications of the Cholesterol Metabolite, 4β-Hydroxycholesterol, as a Sensitive Endogenous Biomarker for Hepatic CYP3A Activity Evaluated within a PBPK Framework
Source: Pharmaceutics. 2024 Sep 30;16(10):1284. doi: 10.3390/pharmaceutics16101284 (PMC11510160; doi:10.3390/pharmaceutics16101284)
Supplement: Supplementary file 1 [file pharmaceutics-16-01284-s001.zip › pharmaceutics-3146506-supplementary.pdf]

SUPPLEMENTARY FILE

Title: Applications of the cholesterol metabolite, 4β-hydroxycholesterol, as a sensitive endogenous biomarker for hepatic CYP3A activity evaluated within a PBPK framework

Authors: Aneesh V. Karkhanis, Matthew D. Harwood, Felix Stader, Frederic Y. Bois, Sibylle Neuhoff

Author Affiliations: Certara UK Limited, Certara Predictive Technologies, Level 2-Acero, 1 Concourse Way, Sheffield, S1 2BJ, UK

Corresponding Author: Aneesh Karkhanis, Certara UK Limited, Certara Predictive Technologies, Level 2-Acero, 1 Concourse Way, Sheffield, S1 2BJ, UK.

aneesh.karkhanis@certara.com

Table of Contents

Methods: 2
- Section 1. Calculation of hepatic CYP27A1 abundance 2

Tables 3
- Table S1. Calculation of Liver Kp of cholesterol and 4β-OHC 3
- Table S2: Final input parameters for CYP3A inducers and inhibitor. 4
- Table S3: Trial design, population characteristics of clinical studies used to verify the predictions of baseline 4β-OHC plasma concentrations in different ethnicities and sexes 9
- Table S4: Trial design, predicted and observed 4β-OHC levels of DDI simulations for the Cholesterol and 4β-OHC model development and verification. 11
- Table S5. Model application: Predicted plasma 4β-OHC concentrations in CYP3A4 and CYP3A5 polymorphic populations, RA and HV populations 12
- Table S6. Model application: Predicted plasma 4β-OHC concentrations in the absence and presence of moderate CYP3A inducers in healthy volunteers and RA patients. 13

Figures 14

References 17

Methods:

Section S1. Calculation of hepatic CYP27A1 abundance

CYP27A1 abundance was determined in clinical liver samples from two White individuals as described in the proteomics study [1]. The average CYP27A1 concentration in tissue homogenate was 14.37 pmol/mg homogenate protein and total homogenate protein was 116.08 mg/g liver. The CYP27A1 abundance was scaled to per mg microsomal protein as follows:

$$CYP27A1\ abundance\ \left(\frac{pmol}{mg}\,mic\ protein\right) = \frac{CYP27A1\ concentration\ \left(\frac{pmol}{mg}\right) \times total\ homogenate\ protein\ \left(\frac{mg}{g}\,liver\right)}{MPPGL\ \left(\frac{mg}{g}\right)} \quad (1)$$

Where, microsomal protein per gram of liver (MPPGL) = 39.79 mg/g liver

The CYP27A1 abundance was calculated to be **41.93 pmol/mg microsomal protein**. Since the data was obtained from two samples, the CV was assumed to 50% in line with other hepatic CYP450 enzymes in default Simcyp library.

Tables

Table S1. Calculation of Liver Kp of cholesterol and 4β-OHC

The Liver kp for cholesterol was calculated from a Turley et al., 1995 study. Cholesterol in liver and plasma was measured in feral adult male cynomolgus monkeys (*Macaca fascicularis*).

**Calculation of Liver Kp values for cholesterol**			
**Parameter**	**Value**	**Units**	**Notes/References**
Cholesterol in monkey liver	4.95	mg/g liver	Page 73 [2]
Monkey Liver weight	90.4	g	Table 1 [2]
Human liver weight	1737.11	g	From default Simcyp V23
total cholesterol in monkey liver = cholesterol * liver weight	447.48	mg	
Total cholesterol in human liver = cholesterol * liver weight	8598.69	mg	Assuming hepatic cholesterol levels are same in monkeys and humans
liver volume	1.60	L	Default value from Simcyp V23
total cholesterol concentration human = amount of cholesterol in liver/(liver volume*1000)	5.35	mg/mL	
Plasma concentration of cholesterol	1.74	mg/mL	Calculated from plasma level reported in [3-5]
**Kp scalar**	**3.07**		Liver/Plasma concentration ratio
**Calculation of Liver Kp values for 4β-OHC**			
4β-OHC amount in liver	21	ng 4β-OHC /mg cholesterol	[3]
4β-OHC in liver = 4β-OHC amount * cholesterol concentration in liver	112.29	ng/mL	
plasma concentration of 4β-OHC	29.85	ng/mL	From meta-analysis
**Kp scaler**	**3.87**		**Liver /Plasma concentration ratio**

Table S2: Final input parameters for CYP3A inducers and inhibitor.

Parameter	Rifampicin	Carbamazepine	Carbamazepine-10,11-epoxide	Phenytoin	Efavirenz	Phenobarbital	Ketoconazole
Mol Weight (g/mol)	823	236.27	252.27	252.28	315.68	232.24	531.4
log P	4.01	2.22	1.44	2.47	4.02	1.47	4.04
Compound Type	Ampholyte	Neutral	Neutral	Monoprotic Acid	Monoprotic Acid	Monoprotic Acid	Diprotic Base
pKa 1	1.7	N.A.	N.A.	8.15	10.2	7.3	2.94
pKa 2	7.9	N.A.	N.A.	N.A.	N.A.	N.A.	6.51
B/P	0.9	1.07	1.53	0.61	0.74	0.83	0.62
fu_p	0.116	0.25	0.48	0.1	0.029	0.49	0.029
**Absorption Model**	First-Order	First-Order	First-Order	First-Order	First-Order	First-Order	First-Order
fa	Predicted (0.98)	0.84 (CV = 30%)	Predicted (0.97)	0.9 (CV = 30%)	0.67 (CV = 15%)	1 (CV = 10%)	1 (CV = 30%)
ka (1/h)	Predicted (1.59)	0.5 (CV = 30%)	Predicted (1.31)	0.53 (CV = 30%)	0.41 (CV = 15%)	2 (CV = 30%)	0.78 (CV = 30%)
Lag time (h)	N.A.	N.A.	N.A.	N.A.	0.36 (CV = 0%)	N.A.	N.A.
fu_{gut}	1	1	1	1	0.005	1	0.06
$P_{eff,man}$ (10^{-4}cm/s)	Predicted from Caco-2 Assay: Apical pH: Basolateral pH – 6.4:7.4 P_{app} (10^{-6} cm/s) – 15 Reference Compound	N.A.	Predicted from Physicochemical properties: PSA (Å^2) – 58.9 HBD - 2	N.A.	N.A.	N.A.	N.A.

	(Propranolol) – 21.15<br>Scaler=1						
**Distribution Model**	Minimal PBPK	Minimal PBPK	Minimal PBPK	Minimal PBPK	Minimal PBPK	Minimal PBPK	Minimal PBPK
Vss (L/kg)	0.42	0.78 (CV = 30%)	0.78 (CV = 30%)	0.57 (CV = 18%)	Predicted - Method 2<br>k_{in} (1/h) – 0.29<br>k_{out} (1/h) – 0.09<br>V_{SAC} (L/kg) – 1.1	0.54 (CV = 30%)	0.345 (CV = 30%)
Kp Scalar	1	1	1	1	0.155	1	1
**Elimination – In vivo clearance**							
CL_{iv} (L/h)	8.7 (CV = 30%)	N.A.	N.A.	N.A.	N.A.	N.A.	N.A.
CL_{po} (L/h)	N.A.	N.A.	6.07 (CV = 36%)	N.A.	N.A.	0.31 (CV = 20.5%)	7.4 (CV = 40%)
CL_R (L/h)	1.26	0.0084	0.14	0.015	0	0.074	0.147
**Enzyme kinetics**							
		CYP3A4:<br>V_{max} (pmol/min/pmol) - 0.72<br>K_m (µM) – 180.2		CYP2C9:<br>V_{max} (pmol/min/pmol) - 0.24<br>K_m (µM) – 4.1	CYP1A2:<br>CL_{int} (µL/min/pmol) - 0.02028		
		CYP3A4:<br>V_{max} (pmol/min/pmol) – 1.44<br>K_m (µM) – 332.3		CYP2C19:<br>V_{max} (pmol/min/pmol) – 1.53<br>K_m (µM) – 36.8	CYP2B6:<br>CL_{int} (µL/min/pmol) – 1.024		

		CYP2C8:<br>V_{max} (pmol/min/pmol) – 0.03<br>K_m (µM) – 741.74		CL_{int} (HLM): (µL/min/mg protein) – 0.97	CYP2A6:<br>CL_{int} (µL/min/pmol) – 0.2387		
		CYP3A4:<br>CL_{int} (µL/min/pmol) – 0.0106			CYP3A4:<br>CL_{int} (µL/min/pmol) – 0.00775		
		UGT 2B7:<br>V_{max} (pmol/min/pmol) – 0.79<br>K_m (µM) – 126.9<br>fu_{mic} – 0.2<br>rUGT Scalar<br>Liver Scalar – 5.29<br>Intestine Scalar – 0.63<br>Kidney Scalar – 0.85			CL_{int} (HLM): (µL/min/mg protein) – 2.39		
		CL_{int} (HLM): (µL/min/mg protein) – 0.255					
**Interaction: Enzymes and transporters**							
CYP1A2	Ind_{max} -2.7<br>$IndC_{50}$ (µM) - 0.1	N.A.	N.A.	N.A.	N.A.	N.A.	N.A.
CYP2B6	Ind_{max} - 5.04<br>$IndC_{50}$ (µM) - 0.07	N.A.	N.A.	Ind_{max} - 10.7<br>$IndC_{50}$ (µM) - 9.8	Ind_{max} - 6.2<br>$IndC_{50}$ (µM) - 1.2<br>fu_{mic} – 0.15	N.A.	N.A.

CYP2C8	K_I (μM) – 24.5<br>Ind_{max} – 6.7<br>$IndC_{50}$ (μM) - 0.3	N.A.	N.A.	N.A.	N.A.	N.A.	K_I (μM) – 2.5<br>fu_{mic} – 0.87
CYP2C9	N.A.	N.A.	N.A.	Ind_{max} – 1.9<br>$IndC_{50}$ (μM) – 15.3	N.A.	Ind_{max} - 3.74<br>$IndC_{50}$ (μM) - 68	K_I (μM) – 10<br>fu_{mic} – 0.95
CYP3A4	K_I (μM) – 15<br>Ind_{max} – 16<br>$IndC_{50}$ (μM) - 0.32	Ind_{slope} - 0.16	Ind_{slope} - 0.14	Ind_{max} – 6.2<br>$IndC_{50}$ (μM) – 7.7	Ind_{max} – 9.9<br>$IndC_{50}$ (μM) – 3.8	Ind_{max} – 23.4 (CV = 30%)<br>$IndC_{50}$ (μM) – 334.9	K_I (μM) – 0.015<br>fu_{mic} – 0.97
CYP3A5	Ind_{max} - 16<br>$IndC_{50}$ (μM) - 0.32	Ind_{slope} - 0.16	Ind_{slope} - 0.14	N.A.	N.A.	N.A.	K_I (μM) – 0.109<br>fu_{mic} – 0.96
UGT1A1	Ind_{max} – 3.16<br>$IndC_{50}$ (μM) - 0.39	N.A.	N.A.	N.A.	N.A.	N.A.	N.A.
P-gp	N.A.	N.A.	N.A.	N.A.	N.A.	N.A.	Gut & Liver<br>K_I (μM) – 0.05

N.A.: not applicable
fu_p: fraction unbound in plasma
fa: fraction absorbed; Ka: absorption rate constant
fu_{gut}: fraction unbound in enterocytes; $P_{eff,man}$: effective permeability in human jejunum
PSA: Polar surface area; HBD: Hydrogen bond donors
Vss: volume of distribution at steady state
CL_{iv}: Intravenous clearance; CL_{po}: oral clearance; CL_R: renal clearance
V_{max}: maximum rate of metabolism; K_m: Michaelis-Menten constant; CL_{int}: in vitro intrinsic clearance;
fu_{mic}: fraction unbound in microsomes (fu_{mic}= 1; unless overwise mentioned)
Ind_{max}: maximal fold induction; $IndC_{50}$: test compound concentration causing half maximal induction

Ind_{slope}: Slope of fold induction vs concentration plot when induction is linear within range of test compound
K_i: concentration of inhibitor that supports half maximum inhibition.
P-gp: P-glycoprotein

Table S3: Trial design, population characteristics of clinical studies used to verify the predictions of baseline 4β-OHC plasma concentrations in different ethnicities and sexes.

Studies reporting 4β-OHC levels in different ethnic populations							
**Study Reference**	**Simcyp Virtual Population**	**Number of subjects**	**Age range (years)**	**Proportion of females**	**Predicted 4β-OHC Css (ng/mL)**	**Observed 4β-OHC Css (ng/mL)**	**P/O**
[6]	NA white	100	45-75	0.36	27.74 ± 20.03	27.47 ± 11.84	1.01
[7]	NA white	10	47-58	0.7	36.82 ± 26.28	31.77 ± 5.04	1.16
[6]	NA Asian	38	45-75	0.39	34.33 ± 25.10	34.47 ± 24.45	1.06
[6]	NA African American	133	45-75	0.48	33.3 ± 24.49	32.1 ± 14.98	1.04
[8]	NA African American	2*	23-41	1	38.05 ± 24.78	30.655 ± 1.62	1.24
[6]	NA Latino	28	45-75	0.35	26.94 ± 19.78	25.34 ± 15.9	1.06
[9]	NEurCaucasian	119*	20-75	0.71	28.99 ± 21.15	32.78 ± 8.92	0.88
[3]	NEurCaucasian	10	45-75	0.38	29.37 ± 21.2	29 ± 10	1.01
[4]	NEurCaucasian	147*	35-75	0.35	29.94 ± 21.73	31.1 ± 13	0.96
[10]	Korean	30	20-40	0.5	30.72 ± 18.86	32.48 ± 14.34	0.92
[11]	Korean	8	22-70	0.5	24.35 ± 14.74	26.83 ± 5.35	0.91
[12]	Japanese	10	27-35	1	36.51 ± 32.84	42.8 ± 13.78	0.85
[13]	Japanese	9	20-32	0.22	23.11 ± 24.54	23.57 ± 7.34	0.98

Studies reporting 4β-OHC levels in males and females							
**Study Reference**	**Population**	**Number of subjects**	**Age range (years)**	**Sex**	**Predicted 4β-OHC Css (ng/mL)**	**Observed 4β-OHC Css (ng/mL)**	**P/O**
[6]	NA white	20	45-75	Males	26.71 ± 18.73	25.1 ± 10.35	1.06
		20	45-75	Females	38.94 ± 23.27	31.6 ± 13.09	1.23
[6]	NA Asian	23	45-75	Males	36.18 ± 24.94	28 ± 10.77	1.29
		15	45-75	Females	49.81 ± 33.73	44.4 ± 34.98	1.12
[6]	NA African American	69	45-75	Males	28.45 ± 20.88	29.8 ± 14.2	0.95
		64	45-75	Females	39.79 ± 24.21	34.6 ± 15.51	1.15
[6]	NA Hispanic-Latino	18	45-75	Males	24.63 ± 16.37	22.7 ± 14.94	1.09
		10	45-75	Females	36.17 ± 24.30	30.1 ± 17.26	1.20
[9]	NEurCaucasian	34	18-75	Males	28.46 ± 23.03	31.67 ± 10.2	0.89
		85	18-75	Females	36.54 ± 25.41	38.99 ± 16.97	0.95
[10]	Korean	15	24-36	Males	27.40 ± 27.19	26.57 ± 10.02	1.03
		15	23-36	Females	40.60 ± 36.13	38.38 ± 15.82	1.05

NA: North American; NEur: North European; mean ± SD; P/O: predicted/observed

*the number of trials for these simulations were adjusted such that 200 individuals were included in the trial population.

Table S4: Trial design, predicted and observed 4β-OHC levels of DDI simulations for the Cholesterol and 4β-OHC model development and verification.

Study Reference	Simcyp Virtual Population	Number of subjects	Age range (years)	Proportion of females	Rifampicin Dosing regimen	Predicted mean± SD Css ratio	Observed mean Css ratio	P/O
[14]	NA white: 0.95<br>NA Asian: 0.05	65	54-64	1	10 mg q.d., 11 days followed by 600 mg q.d., 11 days	3.43±1.28	3.90	0.88
[15]	NEurCaucasian	12	23-52	0.4	600 mg q.d., 15 days	3.54±1.32	3.99	0.89
[16]	HV	16	18-55	0.5	600 mg q.d., 14 days	3.31±1.99	3.32	1.00
[17]	NA white: 0.64<br>NA African American: 0.18<br>NA Hispanic Latino: 0.18<br>HV: 0.09	11	19-48	0	600 mg q.d., 28 days	4.95±1.74	4.84	1.02
[7]	HV	10	46-70	0.7	600 mg q.d., 7 days	2.27±0.85	2.78	0.78
[18]	HV	18	21-50	0.5	600 mg q.d., 8 days	2.50±0.94	2.78	0.90

NA: North American; NEur: North European; HV: Healthy volunteers; mean ± SD; P/O: predicted/observed

Table S5. Model application: Predicted plasma 4β-OHC concentrations in CYP3A4 and CYP3A5 polymorphic populations, RA and HV populations

Simulations	Virtual Ethnic Population	Trial Characteristics	Mean ± SD Css (ng/mL)	5th and 95th CI Css (ng/mL)
CYP3A4 Polymorphisms				
CYP3A4*1/*1 (EM)	North European Caucasian	10 subjects, 20 trials, 20-50 years, 50% females, 700 days	27.91 ± 20.30	6.20, 67.96
CYP3A4*1/*22 (IM)	North European Caucasian		20.59 ± 14.97	4.57, 50.42
CYP3A4*22/*22 (PM)	North European Caucasian		10.55 ± 7.65	2.32, 26.19
CYP3A5 Polymorphisms				
CYP3A5 EM	North American Asian		27.25 ± 17.44	6.94, 59.59
CYP3A5 PM	North American Asian		25.75 ± 16.71	7.27, 61.34
CYP3A5 EM	North American African American		34.05 ± 22.78	8.93, 70.23
CYP3A5 PM	North American African American		32.74 ± 23.75	8.87, 67.21
Rheumatoid Arthritis	North European Caucasian (assumed)	31 subjects, 10 trials, 19-76 years, 80% females, 700 days	16.37 ± 11.31	3.92, 38.85
Healthy Volunteers	HV		29.51 ± 20.85	8.18, 71.51

EM: Extensive metabolizers; IM: Intermediate metabolizers; PM: poor metabolizers; SD: standard deviation; CI: confidence interval.

Table S6. Model application: Predicted plasma 4β-OHC concentrations in the absence and presence of moderate CYP3A inducers in healthy volunteers and RA patients.

Healthy individuals		Baseline 4β-OHC		Induced 4β-OHC		
**CYP3A inducers**	**Dosage regimen**	**Mean ± SD Css (ng/mL)**	**5th and 95th CI Css (ng/mL)**	**Mean ± SD Css (ng/mL)**	**5th and 95th CI Css (ng/mL)**	**Fold induction**
Carbamazepine	600 mg q.d., 400 days	26.21 ± 21.13	7.06, 67.45	59.56 ± 42.27	14.35, 140.31	2.22
Phenytoin	600 mg q.d., 400 days	28.31 ± 21.13	7.04, 68.11	78.29 ± 59.06	13.84, 192.73	2.76
Phenobarbital	100 mg q.d., 400 days	28.38 ± 21.37	6.87, 73.80	79.39 ± 64.50	17.15, 210.27	2.81
Efavirenz	600 mg q.d., 400 days	28.28 ± 21.16	6.87, 73.80	117.42 ± 108.61	21.60, 333.10	4.15
Rifampicin	600 mg q.d., 30 days	29.06 ± 21.12	7.22, 67.43	138.41 ± 98.46	34.88, 314.07	4.76
**RA patients**						
Rifampicin	600 mg q.d., 30 days	16.64 ± 13.94	4.22, 35.70	83.63 ± 68.07	15.15, 217.43	5.02

Css: steady-state plasma concentrations; CI: Confidence interval

Figures

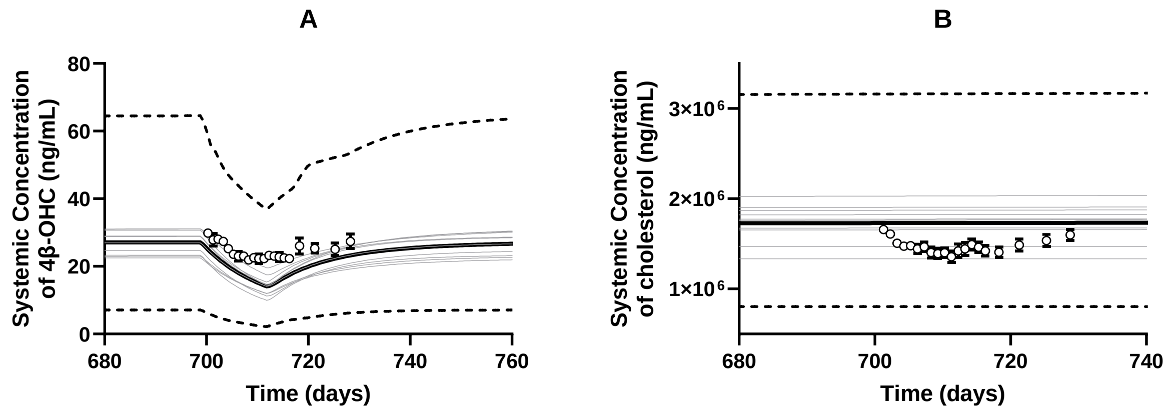

Figure S1. Predicted and observed (open circles) arithmetic mean plasma concentration–time profiles of (A) ketoconazole-driven cholesterol and (B) ketoconazole-driven 4β-OHC levels. Simulations were performed with following trial characteristics: 10 trials × 12 subjects, 20–50 years, a proportion of 5% females, multiple populations (North American Caucasian: North American African American: North American Asian; 0.56:0.41:0.03) with orally administered multiple doses of 400 mg q.d. ketoconazole for 13 days [19]. The black lines represent the population mean plasma concentration–time profiles, the grey lines represent the predictions from individual trials, whereas the dashed lines represent the 5th and 95th percentiles of all simulated subjects' values. Observed data presented as mean ± S.D values according to the Kasichayanula *et al.*, 2014 study [19].

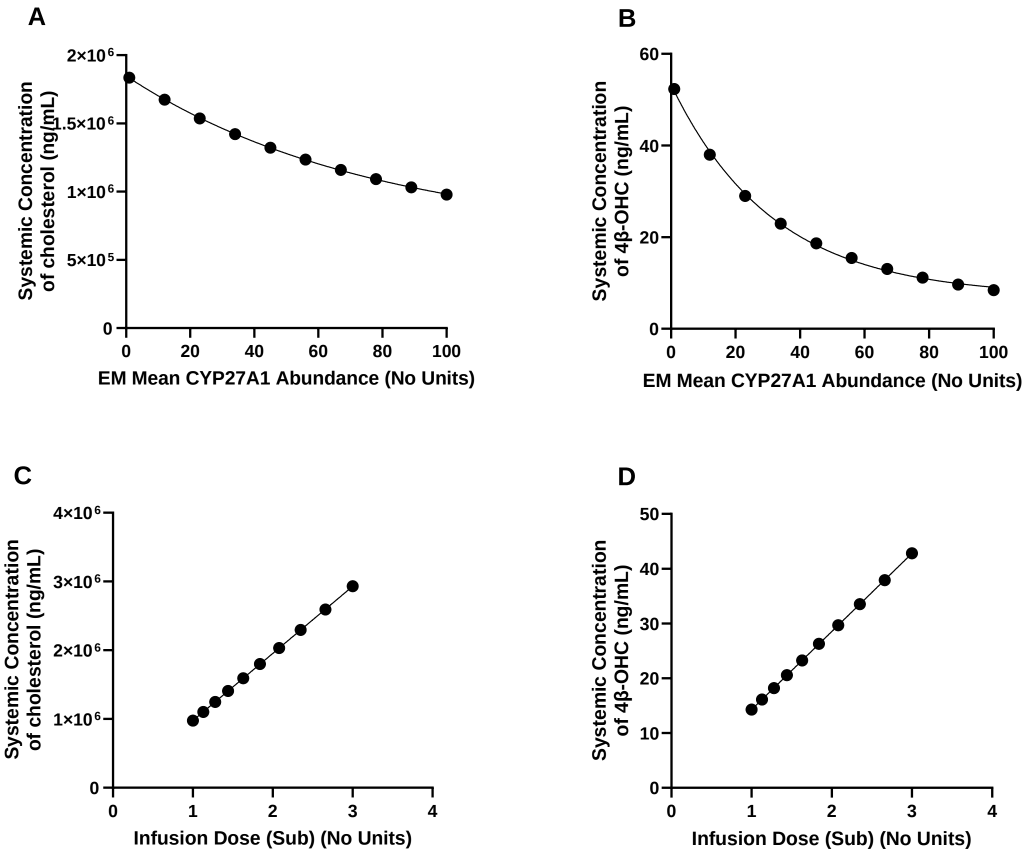

Figure S2. Automated sensitivity analysis was performed to investigate the impact of CYP27A1 abundance and cholesterol dose on (A, C) the predicted cholesterol plasma concentration and (B, D) the predicted 4β-OHC plasma concentration. The simulations showed a linear increase in predicted plasma cholesterol and 4β-OHC concentrations with cholesterol dose. Moreover, a gradual increase in CYP27A1 abundance, decreased cholesterol and 4β-OHC plasma levels.

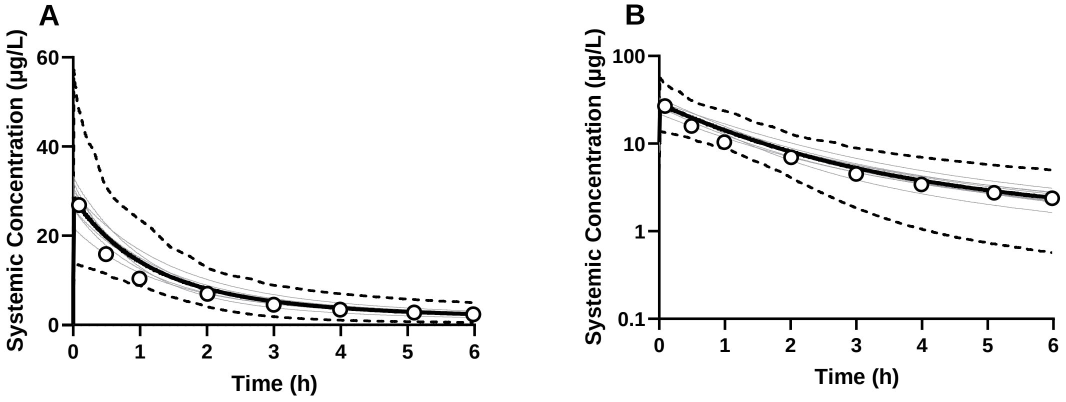

C

PK parameter	Predicted	Observed	Predicted/Observed
C_{max} (µg/L)	26.22	27.5	0.95
AUC (µg*h/L)	45.95	41.2	1.12
$t_{1/2}$ (h)	3.51	3.2	1.09

Figure S3. Verification of reduced CYP3A4 abundance in CYP3A4*1/*22 population with an independent midazolam iv study [20]. Concentration-time profiles in linear (A) and log-linear scale (B), where the grey solid lines, the black solid line, and the dashed black solid lines represent the mean of each trial (10 trials with 12 individuals in each), the population mean, and the 95% confidence interval of all simulated subjects, respectively (n = 120). Predicted and observed pharmacokinetic parameters (C), where Cmax = peak plasma concentration, AUC = area under the curve, and $t_{1/2}$ is the elimination half-life. The model captured the PK parameters within 1.25-fold range.

References

1. Wisniewski, J.R.; Wegler, C.; Artursson, P. Subcellular fractionation of human liver reveals limits in global proteomic quantification from isolated fractions. *Anal Biochem* **2016**, *509*, 82-88, doi:10.1016/j.ab.2016.06.006.
2. Turley, S.D.; Spady, D.K.; Dietschy, J.M. Role of liver in the synthesis of cholesterol and the clearance of low density lipoproteins in the cynomolgus monkey. *J Lipid Res* **1995**, *36*, 67-79.
3. Bodin, K.; Bretillon, L.; Aden, Y.; Bertilsson, L.; Broome, U.; Einarsson, C.; Diczfalusy, U. Antiepileptic drugs increase plasma levels of 4beta-hydroxycholesterol in humans: evidence for involvement of cytochrome p450 3A4. *J Biol Chem* **2001**, *276*, 38685-38689, doi:10.1074/jbc.M105127200.
4. Vanhove, T.; de Jonge, H.; de Loor, H.; Annaert, P.; Diczfalusy, U.; Kuypers, D.R. Comparative performance of oral midazolam clearance and plasma 4beta-hydroxycholesterol to explain interindividual variability in tacrolimus clearance. *Br J Clin Pharmacol* **2016**, *82*, 1539-1549, doi:10.1111/bcp.13083.
5. Hole, K.; Wollmann, B.M.; Nguyen, C.; Haslemo, T.; Molden, E. Comparison of CYP3A4-Inducing Capacity of Enzyme-Inducing Antiepileptic Drugs Using 4beta-Hydroxycholesterol as Biomarker. *Ther Drug Monit* **2018**, *40*, 463-468, doi:10.1097/FTD.0000000000000518.
6. Passarelli, M.N.; McDonald, J.G.; Thompson, B.M.; Arega, E.A.; Palys, T.J.; Rees, J.R.; Barry, E.L.; Baron, J.A. Association of demographic and health characteristics with circulating oxysterol concentrations. *J Clin Lipidol* **2022**, *16*, 345-355, doi:10.1016/j.jacl.2022.03.012.
7. Marschall, H.U.; Wagner, M.; Zollner, G.; Fickert, P.; Diczfalusy, U.; Gumhold, J.; Silbert, D.; Fuchsbichler, A.; Benthin, L.; Grundstrom, R.; et al. Complementary stimulation of hepatobiliary transport and detoxification systems by rifampicin and ursodeoxycholic acid in humans. *Gastroenterology* **2005**, *129*, 476-485, doi:10.1016/j.gastro.2005.05.009.
8. Lee, J.; Fallon, J.K.; Smith, P.C.; Jackson, K.D. Formation of CYP3A-specific metabolites of ibrutinib in vitro is correlated with hepatic CYP3A activity and 4beta-hydroxycholesterol/cholesterol ratio. *Clin Transl Sci* **2023**, *16*, 279-291, doi:10.1111/cts.13448.
9. Bjorkhem-Bergman, L.; Nylen, H.; Norlin, A.C.; Lindh, J.D.; Ekstrom, L.; Eliasson, E.; Bergman, P.; Diczfalusy, U. Serum levels of 25-hydroxyvitamin D and the CYP3A biomarker 4beta-hydroxycholesterol in a high-dose vitamin D supplementation study. *Drug Metab Dispos* **2013**, *41*, 704-708, doi:10.1124/dmd.113.051136.
10. Yoon, S.; Jeong, S.; Jung, E.; Kim, K.S.; Jeon, I.; Lee, Y.; Cho, J.Y.; Oh, W.Y.; Chung, J.Y. Effect of CYP3A4 metabolism on sex differences in the pharmacokinetics and pharmacodynamics of zolpidem. *Sci Rep* **2021**, *11*, 19150, doi:10.1038/s41598-021-98689-z.
11. Oh, J.; Kim, A.H.; Lee, S.; Cho, H.; Kim, Y.S.; Bahng, M.Y.; Yoon, S.H.; Cho, J.Y.; Jang, I.J.; Yu, K.S. Effects of renal impairment on the pharmacokinetics and pharmacodynamics of a novel dipeptidyl peptidase-4 inhibitor, evogliptin (DA-1229). *Diabetes Obes Metab* **2017**, *19*, 294-298, doi:10.1111/dom.12813.
12. Naito, T.; Kubono, N.; Ishida, T.; Deguchi, S.; Sugihara, M.; Itoh, H.; Kanayama, N.; Kawakami, J. CYP3A activity based on plasma 4beta-hydroxycholesterol during the early postpartum period has an effect on the plasma disposition of amlodipine. *Drug Metab Pharmacokinet* **2015**, *30*, 419-424, doi:10.1016/j.dmpk.2015.08.008.
13. Suzuki, Y.; Oda, A.; Negami, J.; Toyama, D.; Tanaka, R.; Ono, H.; Ando, T.; Shin, T.; Mimata, H.; Itoh, H.; et al. Sensitive UHPLC-MS/MS quantification method for 4beta- and 4alpha-hydroxycholesterol in plasma for accurate CYP3A phenotyping. *J Lipid Res* **2022**, *63*, 100184, doi:10.1016/j.jlr.2022.100184.
14. Wiesinger, H.; Klein, S.; Rottmann, A.; Nowotny, B.; Riecke, K.; Gashaw, I.; Brudny-Kloppel, M.; Fricke, R.; Hochel, J.; Friedrich, C. The Effects of Weak and Strong CYP3A Induction by

Rifampicin on the Pharmacokinetics of Five Progestins and Ethinylestradiol Compared to Midazolam. *Clin Pharmacol Ther* **2020**, *108*, 798-807, doi:10.1002/cpt.1848.
15. Dutreix, C.; Lorenzo, S.; Wang, Y. Comparison of two endogenous biomarkers of CYP3A4 activity in a drug-drug interaction study between midostaurin and rifampicin. *Eur J Clin Pharmacol* **2014**, *70*, 915-920, doi:10.1007/s00228-014-1675-0.
16. Einolf, H.J.; Zhou, J.; Won, C.; Wang, L.; Rebello, S. A Physiologically-Based Pharmacokinetic Modeling Approach To Predict Drug-Drug Interactions of Sonidegib (LDE225) with Perpetrators of CYP3A in Cancer Patients. *Drug Metab Dispos* **2017**, *45*, 361-374, doi:10.1124/dmd.116.073585.
17. Stoch, S.A.; Ballard, J.; Gibson, C.; Kesisoglou, F.; Witter, R.; Kassahun, K.; Zajic, S.; Mehta, A.; Brandquist, C.; Dempsey, C.; et al. Coadministration of Rifampin Significantly Reduces Odanacatib Concentrations in Healthy Subjects. *J Clin Pharmacol* **2017**, *57*, 110-117, doi:10.1002/jcph.780.
18. OLUMIANT (Baricitinib) 2017. Clinical Pharmacology and Biopharmaceutics Reviews(s), NDA#207924. Available online: https://www.accessdata.fda.gov/drugsatfda_docs/nda/2018/207924Orig1s000ClinPharmR.pdf (accessed on 25 May 2024).
19. Kasichayanula, S.; Boulton, D.W.; Luo, W.L.; Rodrigues, A.D.; Yang, Z.; Goodenough, A.; Lee, M.; Jemal, M.; LaCreta, F. Validation of 4beta-hydroxycholesterol and evaluation of other endogenous biomarkers for the assessment of CYP3A activity in healthy subjects. *Br J Clin Pharmacol* **2014**, *78*, 1122-1134, doi:10.1111/bcp.12425.
20. Elens, L.; Nieuweboer, A.; Clarke, S.J.; Charles, K.A.; de Graan, A.J.; Haufroid, V.; Mathijssen, R.H.; van Schaik, R.H. CYP3A4 intron 6 C>T SNP (CYP3A4*22) encodes lower CYP3A4 activity in cancer patients, as measured with probes midazolam and erythromycin. *Pharmacogenomics* **2013**, *14*, 137-149, doi:10.2217/pgs.12.202.
